# Surface Manipulation in Cu- and Ag-Based Pre-Columbian Artifacts

**DOI:** 10.1021/acs.accounts.5c00415

**Published:** 2025-09-16

**Authors:** Gabriel Maria Ingo, Cristina Riccucci, Francesca Boccaccini, Marianna Pascucci, Elena Messina, Gabriella Di Carlo

**Affiliations:** Istituto per lo studio dei materiali nanostrutturati, Consiglio Nazionale delle Ricerche, Strada Provinciale 35d, n. 9, 00010 Montelibretti, Rome, Italy

## Abstract

Numerous metal artifacts of exceptional historical and artistic value from the Moche civilization (ca. 450 AD) were unearthed in the tomb of the Lady of Cao (El Brujo, Peru). The tomb yielded the tattooed, mummified remains of a young woman, who was approximately 25 years old at the time of her demise. The rich array of artifacts and insignia of power found within the tomb provides compelling evidence of her elevated status in the hierarchical Moche society. Among the artifacts, the gilded objects and intriguing apparently bimetallic nose ornaments, featuring adjacent gold and silver surfaces, are particularly noteworthy. These artifacts reveal the sophisticated craftsmanship of Moche metalworkers, who expertly produced and worked on Cu- and Ag-based alloys. Moche metalworkers, once they worked and shaped the alloys to a thickness of approximately 100–150 μm, in some artifacts meticulously formed localized, uniform, and thin (roughly 3–5 μm thick) gold- and silver-enriched surface layers by employing etching agents. This process involved the selective depletion of copper from Cu-based alloys and, in some regions, the removal of both copper and silver from a Ag–Cu–Au ternary alloy. The presence of epitaxially grown micrometric silver wires, which resemble the elongated architecture of naturally occurring silver curls, supports the hypothesis of a subtractive surface treatment. These findings demonstrate a pioneering, though empirical, capacity to produce specific Cu- and Ag-based alloys and to select suitable materials for surface manipulation. This capability led to the tailored chemical modification of the outermost layers, resulting in a fascinating monometallic or bimetallic appearance likely imbued with religious, symbolic, or shamanic values. It is noteworthy that the creation of such enthralling artistic masterpieces was uniquely enabled by this ability to manipulate matter at the micro- and nanoscale, combined with the goldsmiths’ artistic creativity.

## Graphical abstract

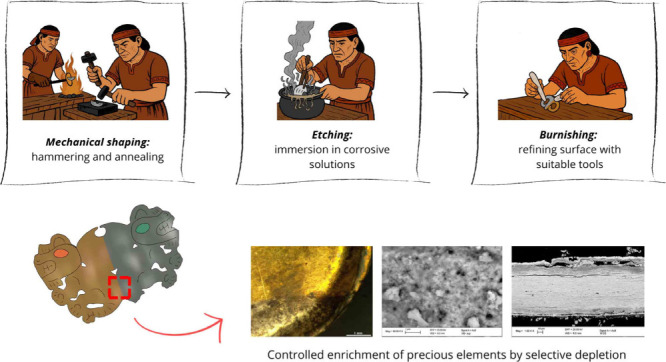

## KEY REFERENCES

-

Ingo, G. M. , Guida, G. ; Angelini, E. ; Di Carlo, G. ; Mezzi, A. ; Padeletti, G. Ancient Mercury-Based Plating Methods: Combined Use of Surface Analytical Techniques for the Study of Manufacturing Process and Degradation Phenomena. Acc. Chem. Res. 2013, 46 (11), 2365–2375.23829823 10.1021/ar300232e^1^ This work mainly focuses on the techniques employed by pre-Romans, Romans, and metallurgists of the Dark Ages to coat with micrometric uniform gold and silver films metal artifacts for both embellishment and counterfeiting.
-

Ingo, G. M. ; Riccucci, C. ; Guida, G. ; Albini, M. ; Giuliani, C. ; Di Carlo, G. Rebuilding of the Burial Environment from the Chemical Biography of Archeological Copper-Based Artifacts. ACS Omega 2019, 4, 11103–11111.31460208 10.1021/acsomega.9b00569PMC6648805 ^2^ By adopting a specific research approach, the naturally grown patinas on archeological bronzes reveal their extensive history of environmental interaction. This research was highlighted in a July sixth article by M. Kaplan in *The Economist*, titled “Chemical Biography” 2019 p 66.
-

Ingo, G. M. ; Riccucci, C. ; Pascucci, M. ; Messina, E. ; Giuliani, C. ; Biocca, P. ; Tortora, L. ; Fierro, G. ; Di Carlo, G. Combined use of FE-SEM+EDS, ToF-SIMS, XPS, XRD and OM for the study of ancient gilded artifacts. Appl. Surf. Sci. 2028, 446, 168–176.^3^ The paper describes the surface morphology and chemical features of the micrometric decorative gold layer masterfully deposited using an amalgam. The results enable also the identification of degradation agents and products naturally formed during prolonged interaction with the burial environment.
-

Ingo, G. M. ; Riccucci, C. ; Faraldi, F. ; Pascucci, M. ; Messina, E. ; Fierro, G. ; Di Carlo, G. Roman sophisticated surface modification methods to manufacture silver counterfeited coin. Appl. Surf. Sci. 2017, 421, 109–119.^4^ The paper reveals how Roman metallurgists successfully produced counterfeit coins using sophisticated microplating methods combined with tailored surface chemical modification based on the mercury–silvering process. These findings collectively indicate the possible driving forces behind the forgery production.

## Introduction

In 2006, a team of Peruvian archeologists led by Franco Jordan unearthed the tomb of a high-ranking Moche priestess or female ruler beneath the northwest courtyard of the Huaca Cao Viejo temple, a few hundred meters from the Pacific Ocean (El Brujo, Peru).^5–8^ The tomb contained the tattooed, mummified remains of a young woman, who was about 25 years old. Artifacts and insignia of power found within the tomb indicate her high status in the hierarchical Moche society. The Lady’s death is estimated to have occurred around 450 AD, roughly midway through the Moche civilization’s period of flourishing (circa first to eighth centuries AD) in the arid northern coastal oasis river valleys situated between the Andes and the Pacific Ocean.^5–8^

The discovery of this pre-Inca tomb ranks among South America’s most significant recent archeological finds, notable as one of the few major unlooted graves excavated intact, similar to the royal tombs of the Lords of Sipán.^9,10^ The exceptionally well-preserved mummy was wrapped in vegetable fibers and approximately two dozen cloths, with some areas sprinkled or decorated with red cinnabar (HgS).^7,8^ Within the funerary bundle, nestled between the cloths and close to the body, archeologists discovered numerous spectacular ceremonial metal ornaments, insignia of power, and jewelry, all in generally good condition.^5–8^ The opulence of these objects corroborates the idea that the buried woman was one of the most powerful women in ancient Peru, now known as the Lady of Cao.

These artifacts are not only of exceptional artistic value but also of great importance due to their precious and symbolic nature. They highlight the extraordinary craftsmanship of the Moche artisans, who were skilled in producing high-quality metalworks, often concurrently gilded and silvered. This proficiency places them on par with other exquisite ancient artifacts from Asia and Europe.^1,3,4,11–26^

Among the noteworthy artifacts are ceremonial objects, many of which appear to be made of precious metals, including nose ornaments, known as *narigueras*. These intricately designed artifacts, intended to be hooked into the nostrils, are aesthetically remarkable in many artifacts for their bimetallic appearance, created by the juxtaposition of gold and silver, a duality observed in other Moche ceremonial objects.

The fabrication methods employed in the creation of the *narigueras* were different, with some appearing to have been created using a single sheet of metal, while others exhibit a more complex construction consisting of previously shaped sheets of different metals joined together either mechanically or by some other method.^25,27–29^

Many of the metal funerary objects of the Lady of Cao have been characterized *in situ* using a portable X-ray fluorescence (XRF) instrument, complemented by X-ray radiographic imaging.^27–29^ Subsequently, microchemistry and corrosion products of several selected artifacts, including an intriguing *nariguera*, were analyzed by using scanning electron microscopy (SEM) with energy dispersive spectroscopy (EDS). Some results mostly related to the corrosion products have been reported in a recent publication with a preliminary understanding of how Moche goldsmiths produced the artifacts.^25^ This study presents novel findings from a comprehensive surface and bulk analysis of a selection of symbolic artifacts of the Lady of Cao, including a single-sheet apparently bimetallic *nariguera*. The artifacts were chosen based on an intuitive perception of sophisticated surface manipulation of Au–Cu and Ag–Au–Cu alloys, with the aim of revealing the advanced ingenuity and empirical skill with which the Moche craftsmen worked and manipulated metals. A thorough examination of several artifacts was conducted *in situ* to identify the presence of soldered or welded joints or mechanical connections between the substrate and areas identified as silver or gold. However, no such joints or connections were found in the artifacts examined here, particularly in the selected *nariguera* species studied.

It is important to emphasize that the surface and bulk characteristics of several significant fragments have been analyzed in detail for the first time. We used an attentive investigation approach that can reveal many aspects of the manufacturing techniques and the extensive history of the artifact-environment interaction.^2,4^ This tailored approach is based primarily on the combination of high spatial resolution field emission (FE) SEM using both backscattered (BSE) and secondary electrons (SE) and energy-dispersive X-ray spectroscopy (EDS). The rationale behind selecting these analytical techniques stems from the fact that FE-SEM and EDS provide significantly more accurate microchemical, structural, and morphological results than X-ray fluorescence (XRF) or other low spatial resolution methods.^1–4,27–29^

The recent focus on expanding our understanding of ancient manufacturing technologies has been further fuelled by the results of micro- and nanochemical investigations, which have revealed sophisticated ancient methods involving the manipulation of materials at both micro- and nanoscales.^1–4,30–32^ These findings offer valuable insights into the surface modification of alloys or ceramics intended for the fabrication of new structures and can contribute to design and select reliable conservation materials and methods for preserving these precious artifacts for future generations.^33–35^

## Microchemical Structure of the Artifacts

Visual and optical *in situ* inspection facilitated the selection of artifacts whose surfaces appear to have been modified by Moche metalworkers to alter their appearance. The first object whose surface microstructure is described here is an *atlatl* (Figure 1a), a spear thrower used for hunting and personal defense by pre-Columbian civilizations. Metal *atlatls* could also be used as symbols of rank by Moche people of higher social status, as in this case.

**Figure 1:**
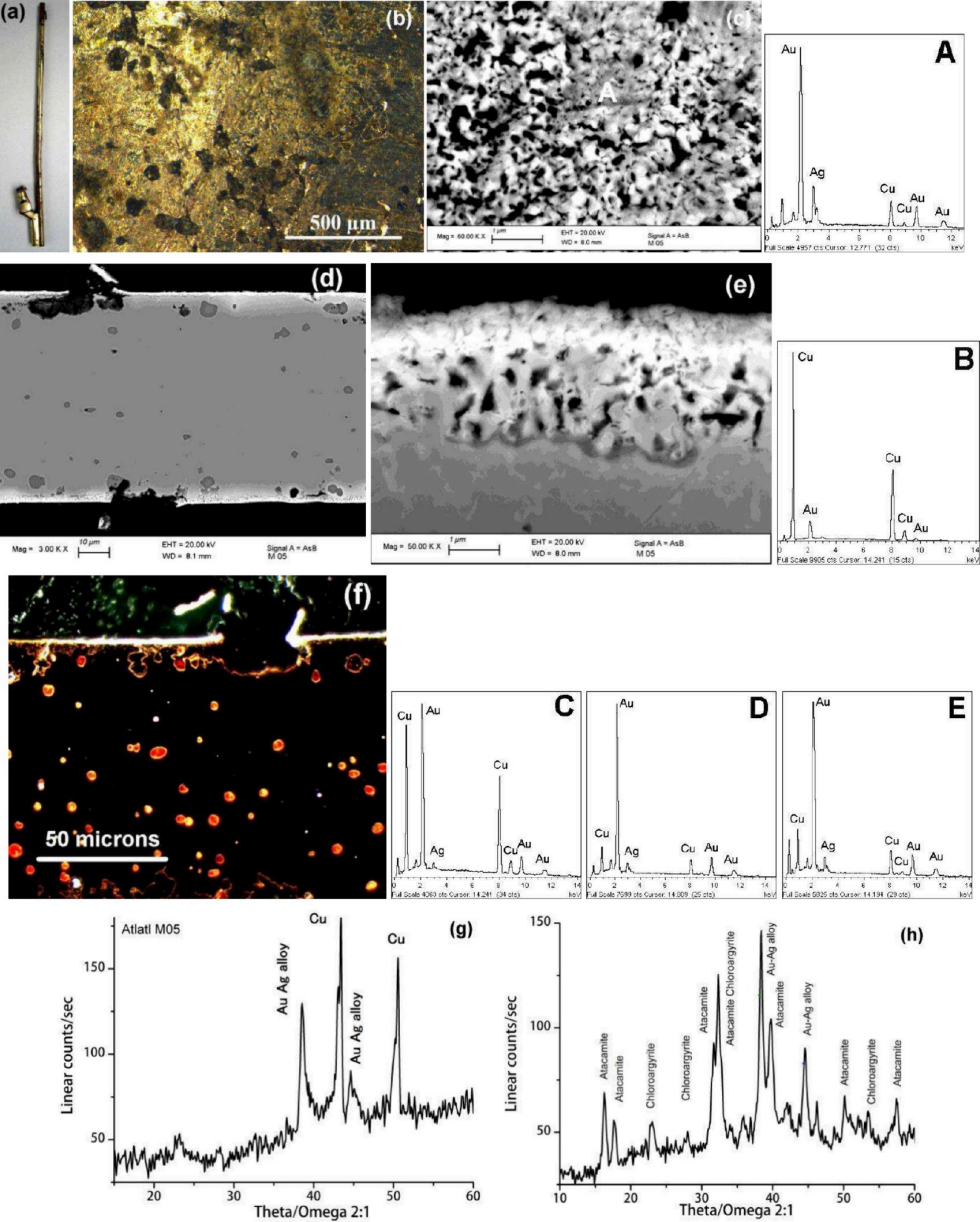
Image (a): a view of the *atlatl* that appears to be made of gold. MO and BSE-FE-SEM images (b) and (c) describes the golden surface morphology. The corresponding chemical composition is provided by the EDS spectrum A. The BSE-FE-SEM micrographs (d) and (e) and the MO image (f) show the structure of a cross-sectioned fragment, and the EDS spectra B, C, D and E reveal the local chemical composition. The XRD patterns of the outermost golden layer and the greenish areas are also shown in (g) and (h), respectively.

As shown by the optical microscopy (MO) image (Figure 1b) the *atlatl* exhibits a golden surface with minor, sporadic zones of dark greenish copper corrosion compounds, suggesting a multimaterial nature. BSE-FE-SEM image (Figure 1c) and EDS spectrum A describe the morphology while bulk structure and thin gold-enriched outermost layer are shown in the BSE-FE-SEM and OM images (Figure 1d–f) supported by the EDS spectra (Figure 1B–E). Finally, the X-ray diffraction (XRD) pattern identifies the surface crystalline species, including corrosion products (Figure 1g,h).

The results demonstrate the presence of an Au-enriched surface layer, the chemical composition of which was determined by EDS. The analysis of the outermost layer (punctual) and of the substrate (analyzed area 50 μm × 70 μm) is as follows: Cu, 28 wt % (hereafter wt %); Au, 65 wt %; Ag, 7 wt % and Cu, 91 wt %; Au, 7 wt %; Ag, 2 wt %, respectively.

The EDS data, combined with the microstructural findings demonstrate how Moche metalworkers manipulated the *atlatl*’s surface, depleting copper to enrich the surface with the gold inherently present in the alloy.^12–17^ The Moche artisans employed a method involving manipulation of the alloy’s surface chemical composition through one or more cycles. Following mechanical shaping, the initial step was likely thermal treatment of the alloy in air, which resulted in the conversion of the surface copper into copper oxides. Subsequently, a corrosive solution was used to selectively remove the copper oxides, leaving a gold-enriched sponge *in situ*. This sponge was then gently compacted and burnished using appropriate tools.^1,3,4,11,12,17,24,36,37^

The removal of the less noble metal, Cu, was probably carried out by immersing the artifact in pickling solutions containing corrosive substances such as plant extracts, ferric or alkaline ferric sulfates capable of removing surface copper oxides.^11–13,24^ The findings of this study corroborate observations made by Lechtman and her associates, they previously noted that Moche metalworkers adeptly employed depletion gilding at a minimal gold concentration within the alloy (in this case about 7 wt %), a level at which a resilient gold-enriched surface layer can be established.^11^

The term *tumbaga* was coined by the Spanish conquistadors to denote these generic alloys, which comprise predominantly Cu and Au, with the latter metal’s content typically less than 20 wt %. *Tumbaga* alloys often contain a variable small amount of Ag, which is a common component of native gold or may have been intentionally added. The *tumbaga* alloys were used by pre-Columbian civilizations in South and Central America to produce significant artifacts with a high surface gold content, likely for symbolic, social, religious, artistic reasons, or other as-yet-unexplained purposes.

The results indicate that the *atlatl* under investigation was produced using a Cu-based alloy with a truly minimal percentage of precious metals and that its chemical nature underwent substantial surface modification to create the illusion of a solid gold artifact.^25^

It is noteworthy that the production of gold- or silver-plated artifacts by pre-Columbian civilizations was not fraudulent, as was sometimes the case in the Old World.^1,3,4,11–13,21–23,30^ The objective of the Moche metalworkers was not to deceive, but rather to modify as appropriate the color and aesthetic appearance of some symbolic or emblematic objects.^1,11–13,21–23,25,37^

X-ray photolelectron spectroscopy (XPS) and EDS were also used to ascertain the surface presence and potential contribution of other minor elements, including As, Hg, Sn, Sb, Zn and Pb.^1,13,16–21,37^ The XPS results (not shown) excluded the presence of these elements on the outermost layers.^8–42^ Furthermore, the EDS investigation revealed that As, Hg, Sn, Zn, and Pb were not present in the bulk alloy, indicating that these metallic elements are not involved in the production of the artifact under study or its surface-enriched layer. As discussed below, our EDS investigation occasionally revealed only the presence of small particles containing mercury, always associated with sulfur. This occurrence is reasonably related to the presence of cinnabar, which was used by Moche as a pigment to decorate the artifacts (Figure S1, Figure S2) and cloths, or sprinkled on the deceased in powdered form during burial.^7,8,20,43^

Regarding the presence of aggressive agents, XPS results confirm the presence of hazardous chloride compounds, such as copper chloride or oxychlorides on some small degraded areas,^25,44–46^ consistent with the XRD results (Figure 1h). The binding energy (BE) of the Cu 2p_3/2_-Cu 2p_1/2_ signals and their line shape analysis including the shakeup satellite reveal the predominant presence of a Cu^2+^ with a probable small contribution of Cu^1+^, likely as cuprite (Cu_2_O).^38,39,44–46^ These results, combined with the OM and FE-SEM-EDS data, confirm the good state of preservation. However, the presence of the undesirable atacamite CuCl_2_·3Cu­(OH)_2_ (Figure 1h), albeit in small quantities, suggests that these degradation products should be removed by tailored cleaning and conservation procedures to ensure their preservation for future generations.^33–35^

The study of the *narigueras* (Figure 2, Figure 3, Figure 4) further highlights the great ability of the Moche metallurgists to manipulate the surface of Cu–Au–Ag ternary alloys. Notable is their intriguing bimetallic appearance characterized by the harmonious juxtaposition of uniform gold and silver areas, distinctly separated by a pronounced color change from Au to Ag. Silver and gold areas are symmetrically present on both the anterior and posterior surfaces of some *narigueras*, including the ornament under study. However, the anterior surface is smooth, shiny, and reflective, in stark contrast to the posterior surface’s wrinkled texture (Figure 3). The symmetry observed in the tooled depressions and thin edges of the studied artifact further supports the hypothesis that these areas were subjected to silvering and gilding processes, rather than to leaf gilding.^1,21–23^

**Figure 2:**
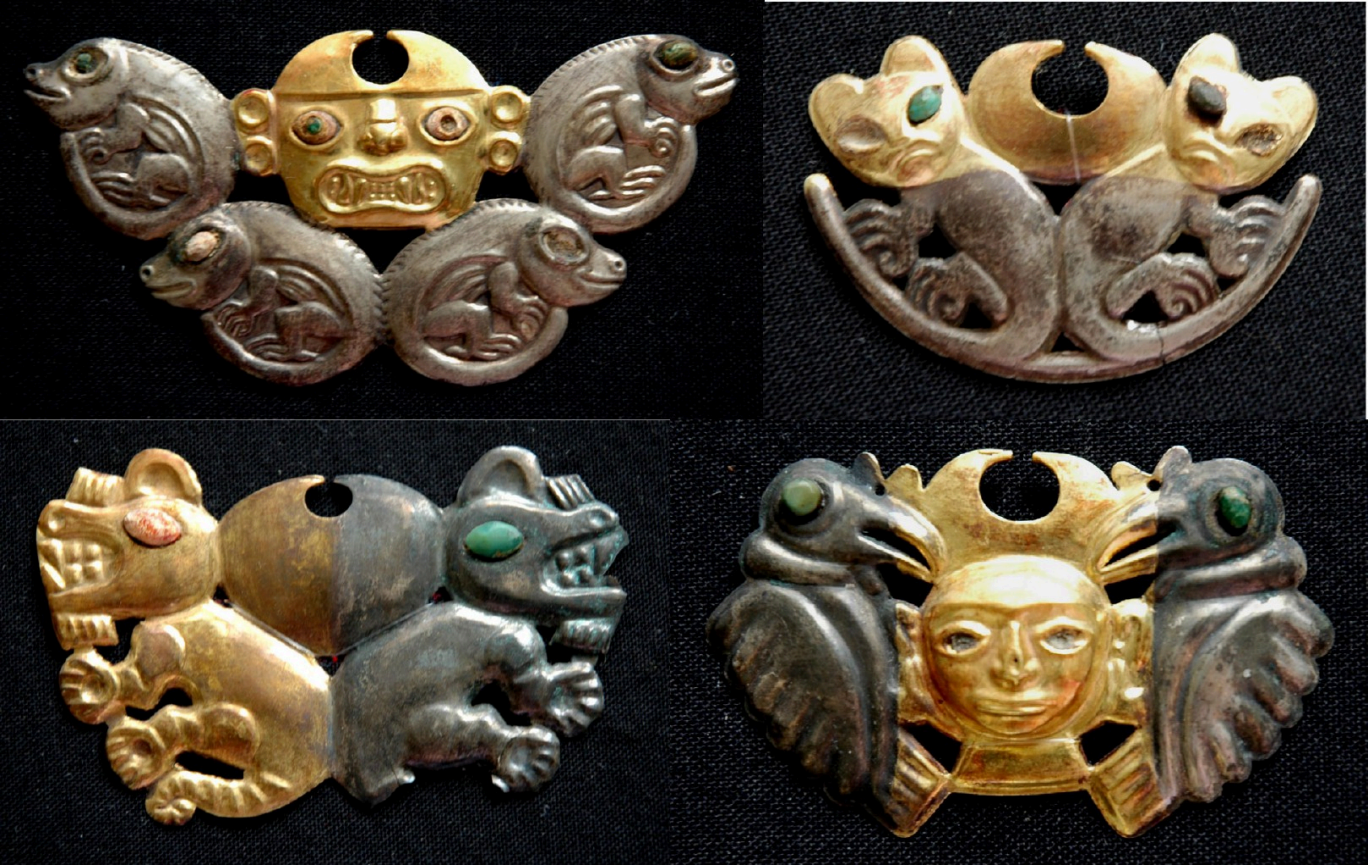
Front view of some *narigueras* found in the tomb of the Lady of Cao. These artifacts are characterized by a clearly visible bimetallic appearance with sharply separated gold and silver areas.

**Figure 3:**
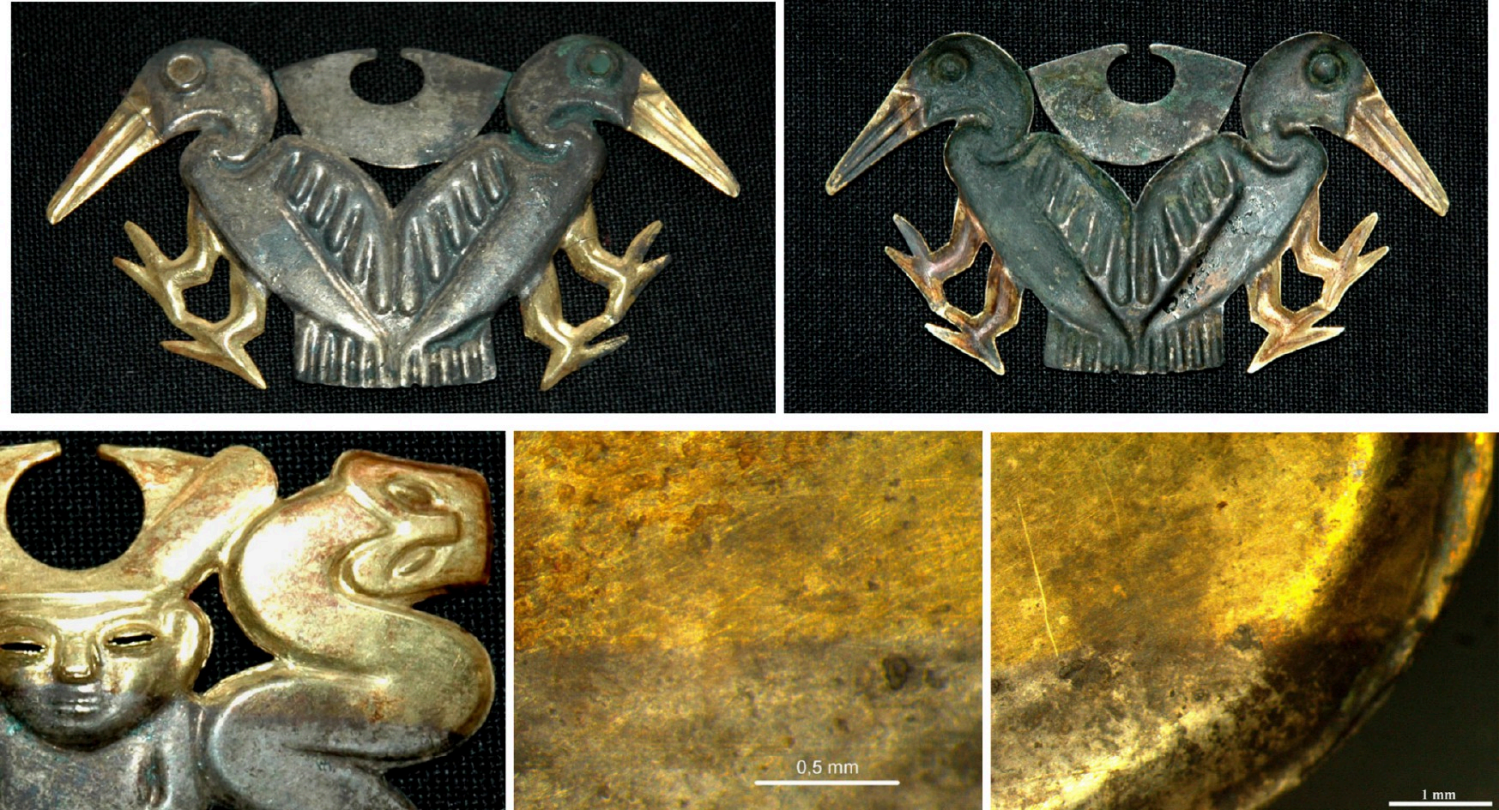
OM images show the front and back of a *nariguera* (left and right, respectively, first row). Note the symmetrical gold areas on both the front and back-side. The front surface appears smooth and reflective, while the back surface is quite wrinkled. The images in the second row show details of the interface between the gold and silver areas in another apparently bimetallic *nariguera* that features gold and silver areas on both sides.

**Figure 4:**
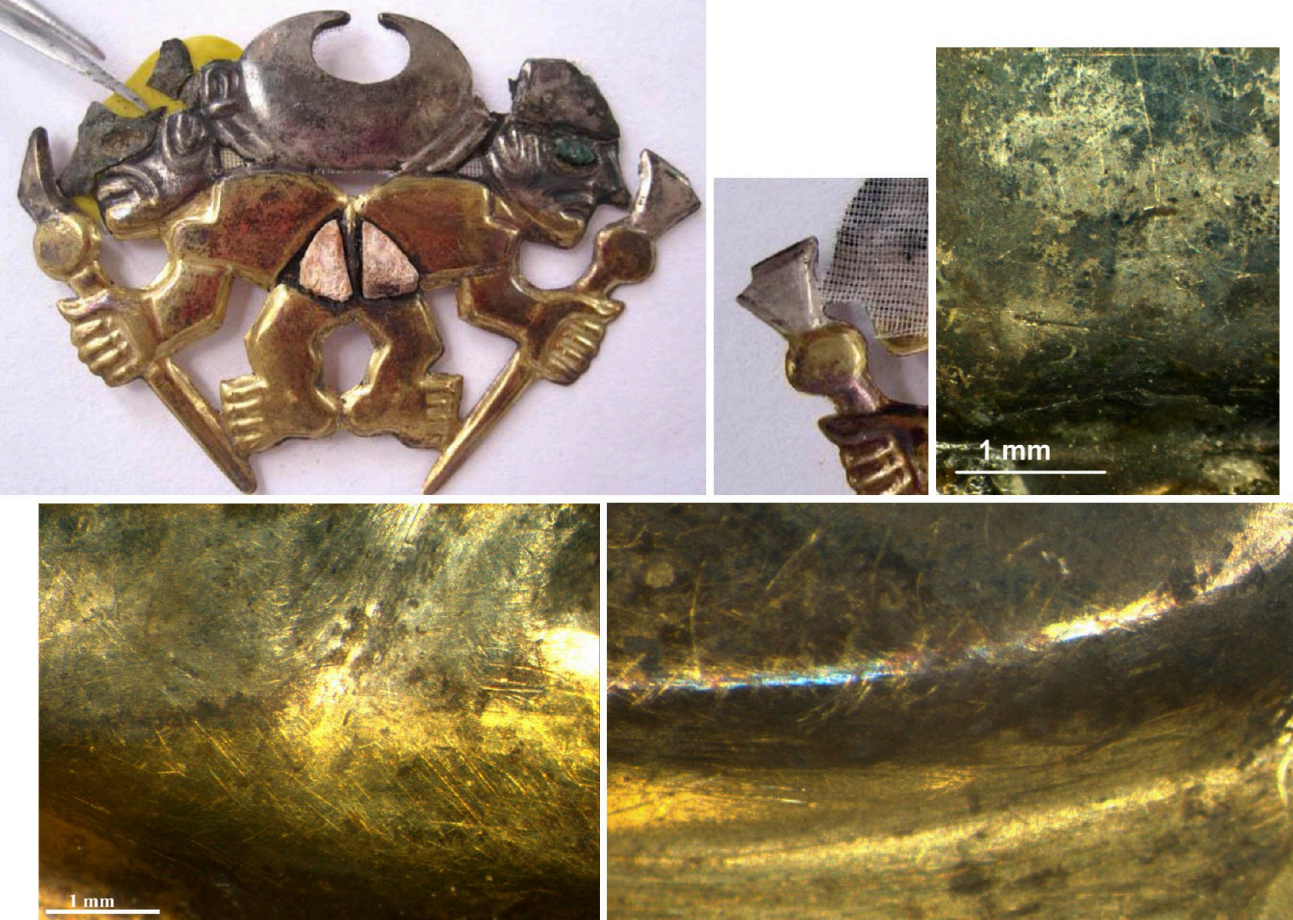
Front of a partially fragmented *nariguera* (on the left, first row). The center image details the back side, showing the gold and silver areas; the right image is an OM micrograph of the burnished silver-plated front of the *nariguera*. The second row images show the sharp interface between gold and silver areas.

The above presented observation implies that the surface manipulation processes involved immersing the embossed object in a suitable pickling solution or poultice containing corrosive chemical species such as salt and ferric sulfate, or urine, with ammonia being one of the decomposition products, compounds available to Andean metalworkers.^11–13^ To obtain more precise details on the micromorphology and local chemical composition, a thorough BSE- and SE-FE-SEM-EDS analysis was conducted on the front side of the silver-plated surface of a *nariguera* fragment (Figure 4) of particular interest as no soldering or mechanical bonds were observed between the Ag or Au areas and the substrate.

The BSE-FE-SEM micrograph shown in Figure 5 (image a) reveals the presence of nanopits, indicative of a chemical manipulation process.^47,48^ This process is hypothesized to have begun with a thermal treatment to form the oxides of the less noble metal(s), namely, copper or copper and silver. The oxides were subsequently removed by suitable solutions, the precise identities of which have yet to be determined. For effective etching of the less noble atoms, the reaction products generated by the pickling solution needed to be dissolved rather than precipitated at the metal–solution interface. Therefore, the Moche artisans likely avoided the presence of sulfide anions. The progressive removal of one or more alloy components resulted in a small volume reduction in the outer layer, leading to the observed open pore structure with nanochannels, nanoteats and lattice defects.^47,48^ The final burnishing process only partially removed these defects and may have created by fragmentation the silver particles whose morphology and chemical composition are shown in Figure 5a,b and EDS spectrum B.

**Figure 5:**
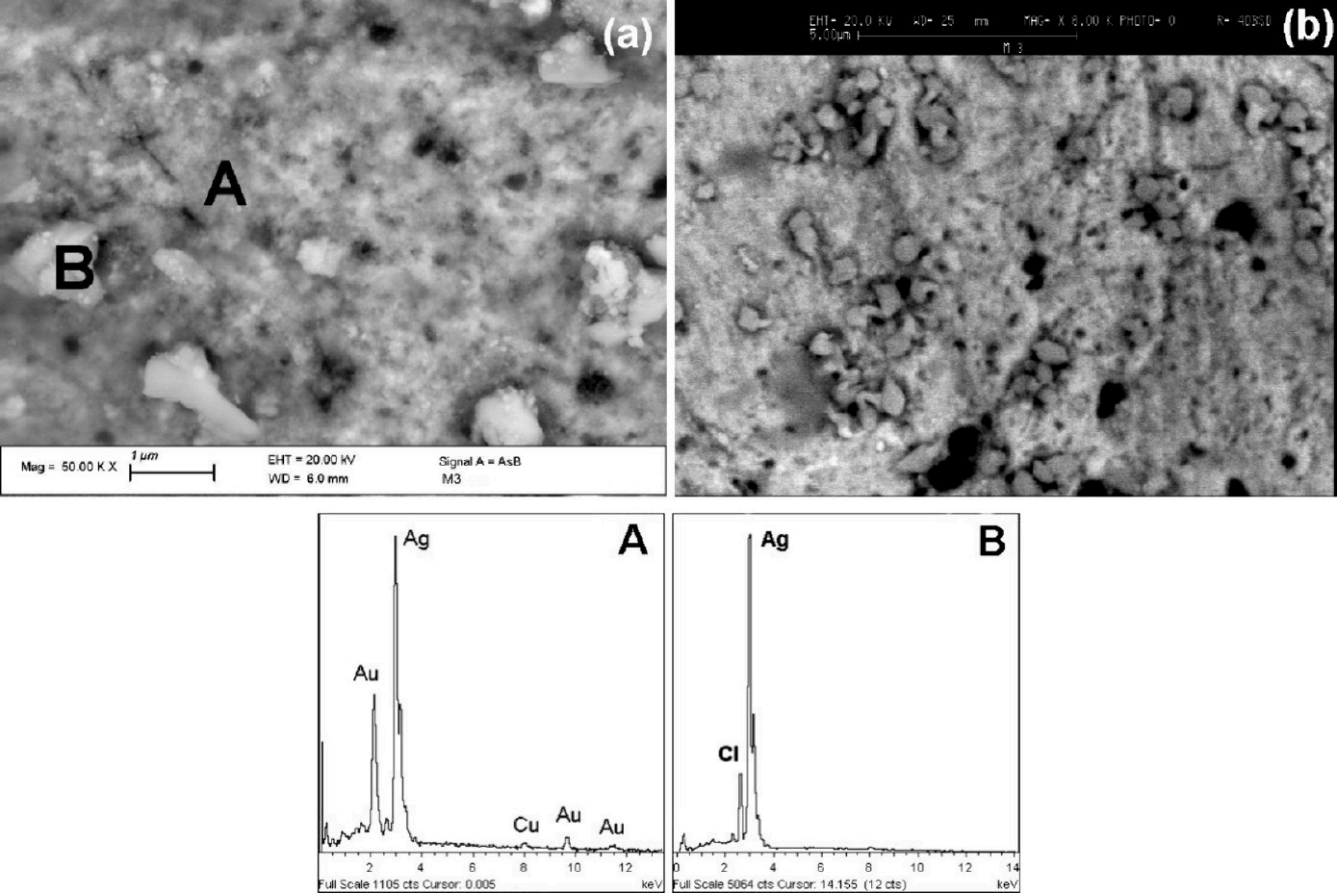
BSE-FE-SEM and BSE-SEM images and EDS spectra of the *nariguera*’s silver front surface showing its morphology and chemical composition.

As proposed by Forty and Durkin, the etching of precious metal-based alloys forms an initial, disordered outer layer. The ordering of this layer is driven by the surface diffusion of the remaining nobler atoms.^47,48^ This self-organization process causes precious metals to diffuse along the step edges, where they continuously accumulate and rearrange. Ultimately, this results in the local formation of island nuclei that grow and merge to form a thin, spongy layer or epitaxial structures with an increased noble metal content. These structures can then be compacted by the metalworkers through a final burnishing.^1,3,22,23,26^ The depletion of the less noble metals does not occur exclusively on the outermost surface but may also occur at a certain depth, even of a few microns.^49,50^

The resulting surface chemistry and structure of the manipulated layer depend on the metallurgical characteristics and chemical composition of the alloy as well as other relevant key factors, including the nature of the etching materials and the technical procedures used. The subtractive method developed by the Moche artisans has the potential to produce an Ag- or Au-enriched layer, the composition and architecture of which can be varied by repeating and/or modifying the removal conditions one or more times, as in modern nanofabrication processes.^49^

Further detailed information was obtained from the BSE-FE-SEM, EDS, and OM investigation of the *nariguera* cross-section whose results are shown in Figures 6 and 7. The findings demonstrate the presence of an outermost silver-enriched thin film on both sides. The layer on the burnished front side is compact and approximately 3–5 μm thick while the silver-enriched layer on the back side exhibits a granular-like structure (Figure 6b, Figure 7a, Figure 7c). BSE-FE-SEM image (Figure 6a) and EDS spectra A and B describe the alloy heterogeneity which is primarily composed of a two-phase structure with varying amounts of Ag, Cu and Au.^50,51^ Furthermore, a comparison of the BSE-FE-SEM images and EDS spectra of the silver-enriched outermost layer and the inner area reveals differences in microchemical composition and metallurgical features (Figures 6a and 7b) which can only be accounted for by a surface manipulation process.

**Figure 6:**
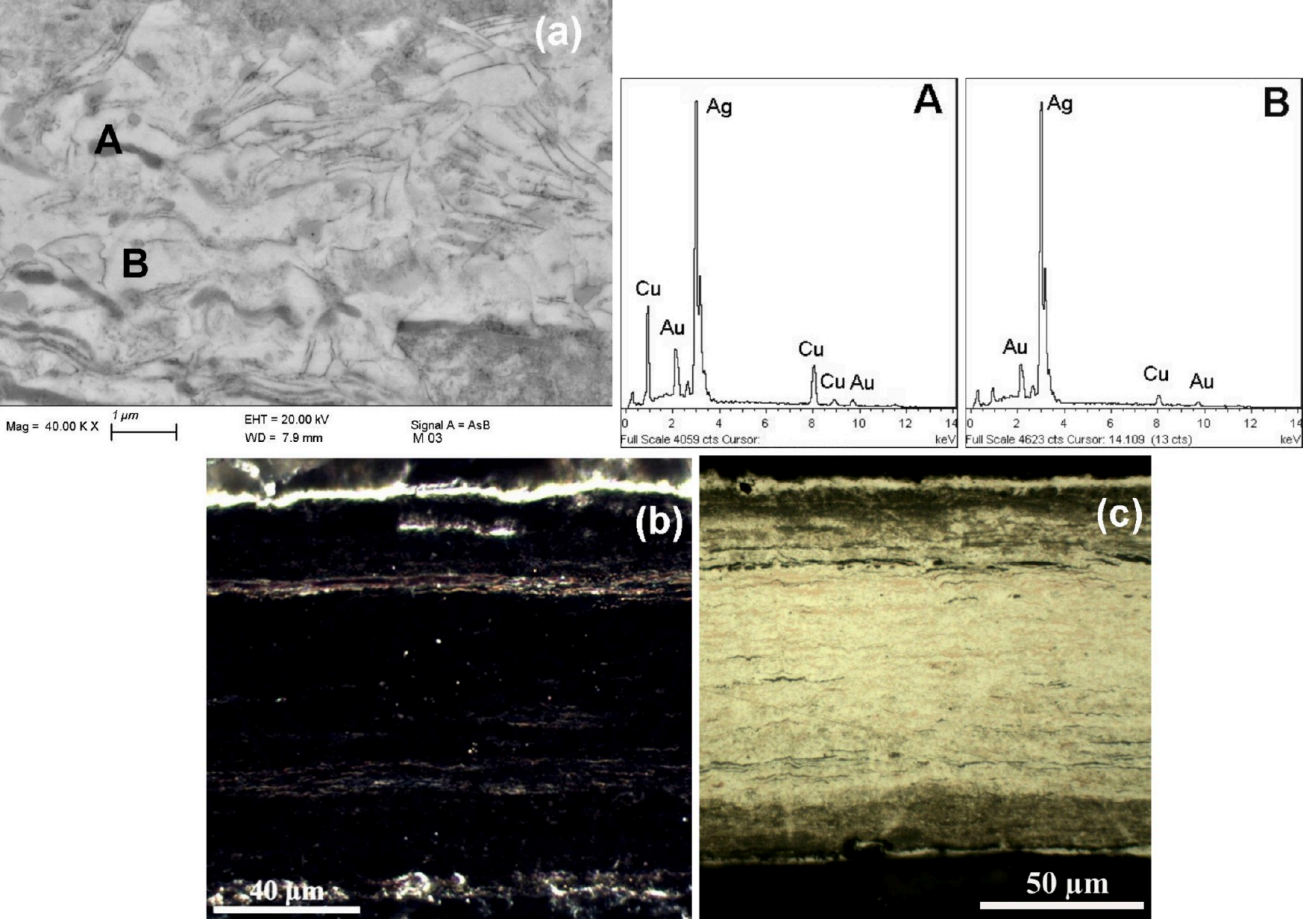
(a) BSE-FE-SEM image and EDS spectra A and B of the sectioned *nariguera* in the bulk area revealing the alloy’s metallurgical features. The Ag-based alloy is composed of chemically distinct phases with varying elemental contents: spectrum A corresponds to a copper-enriched phase, while spectrum B corresponds to a silver-enriched phase. The dark-field OM image (b) shows the presence of a thin layer of reddish cuprite (Cu_2_O) beneath the Cu-depleted region (front side). Furthermore, a thinner surface silver enrichment with a granular-like structure is present on the back side. The bright-field OM image (c) shows pinkish elongated phases with a consistent copper content relative to the surrounding Ag-based matrix.

**Figure 7:**
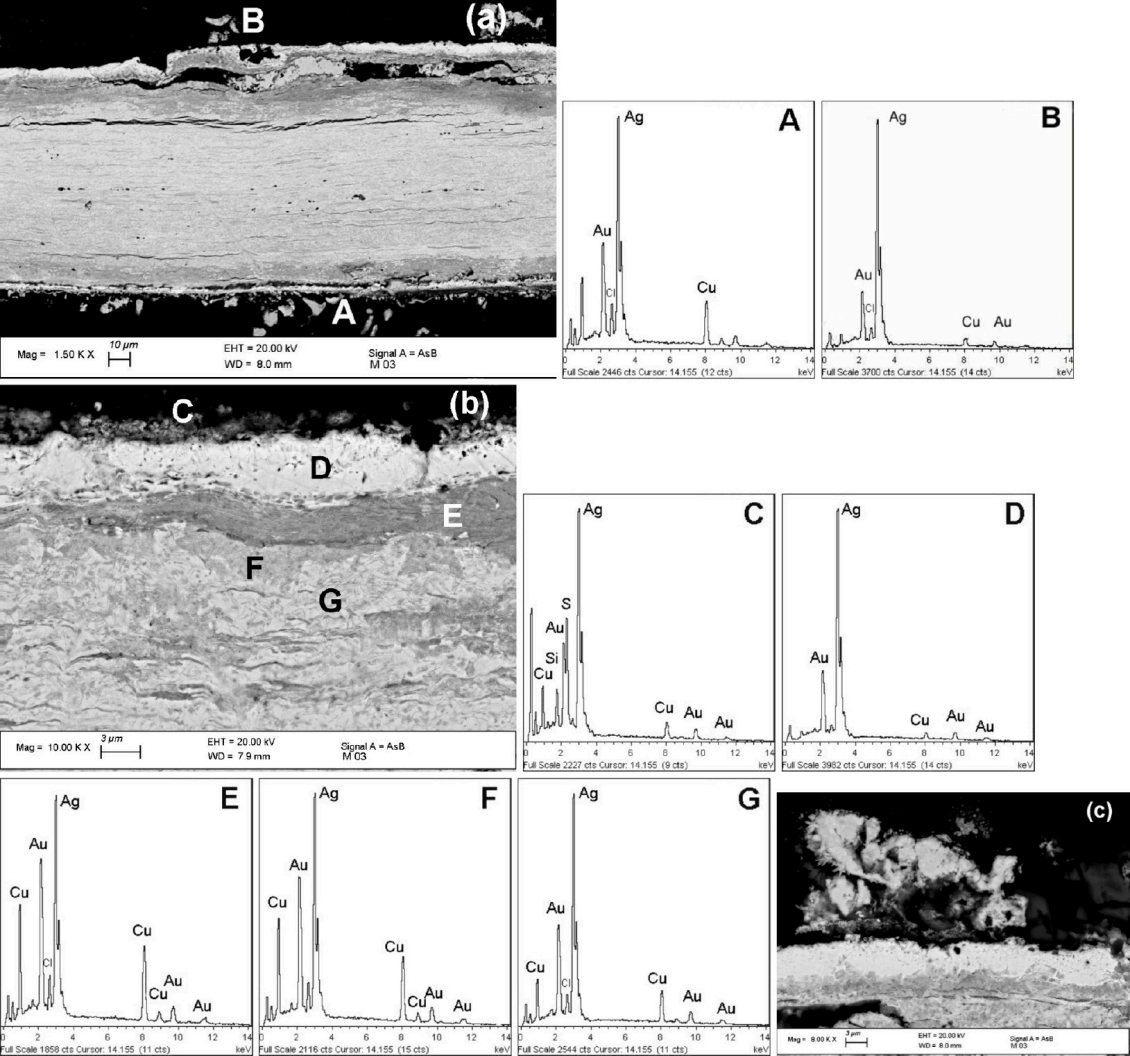
BSE-FE-SEM image (a) and EDS spectra for the cross-sectioned *nariguera* with EDS spectra A and B describing the outermost layer of the unburnished and burnished sides, respectively. The BSE-FE-SEM image (b) reveals microchemical details of the structure of this outermost silver-enriched layer whose thickness is about 3–5 μm. The micrograph (b) and image (a) shown in Figure 6 reveal the differences between the outermost silver-enriched area and the bulk region. Image (c) gives micromorphological details of the granular-like surface structure on the back side.

Other information can be achieved from the dark-field OM image (Figure 6b) which shows the entire cross-section of the *nariguera* and reveals also the presence of a thin layer of reddish cuprite (Cu_2_O) indicating a mild, selective oxidation of the less-noble copper that likely occurred during alloy heating. The bright-field OM image (Figure 6c) confirms the presence of pinkish elongated phases with a consistent copper content. Additionally, the results confirm the exclusion of mercury gilding or silver leaf coating processes.^1,4,27–29^

The EDS quantitative results obtained from the bulk alloy (analyzed area 40 × 40 μm) are shown in Table 1 and demonstrate that the Moche artisans skillfully used a Ag-based ternary alloy to manufacture this *nariguera*, exhibiting the following composition: Ag, 71 wt %; Cu, 18 wt %; Au, 11 wt %. The relatively consistent gold content could indicate deliberate addition beyond what would be expected as a silver impurity.^11–13,16^ Copper was also likely added intentionally, probably to improve the mechanical properties of *nariguera*, which is about 120 μm thick.

**Table 1:** Semiquantitative EDS Data from the Spectra A–G shown in Figure 7 ^a^

Spectrum	Ag (wt %)	Cu	Au	O	S
A	82	6	12		
B	79	2	19		
C	61	5	19	0	4
D	81	2	17		
E	37	30	28	4	
F	64	13	20	2	
G	80	10	10		
Alloy chemical composition	71	18	11		

a The table also reports the overall alloy chemical composition measured via EDS on a large (40 × 40 μm) central area of the artifact.

Regarding the bulk alloy’s different phases, EDS spectra show that the elongated gray areas are richer in copper (Figure 6a and EDS spectrum A), while lighter phases contain more silver (EDS spectrum B). This information reveals a complex bulk structure of narrow, alternating, interlayered copper- and silver-rich phases. These phases are oriented parallel to the surface in a direction consistent with severe mechanical cold or hot working. The structure’s formation is attributed to two factors: the solidification process of a Ag–Cu–Au alloy, in which the miscibility of its constituents created phases with variable metal content, and the subsequent mechanical working and shaping.^15,16,52,53^ The observed elongation of the phases indeed indicates that the alloy underwent significant deformation from a hammering process used to reduce its thickness and shape the *nariguera*. Consequently, the mechanical working of the heterogeneous alloy led to the formation of parallel and elongated phases, with their structure being only minimally altered by the subsequent annealing conducted to restore ductility. It is postulated that, following the repeated application of hammering and annealing, the surface copper content decreased due to the selective oxidation and spalling. This, in turn, favored the subsequent etching process which further reduced the copper or copper and silver content from the outermost region, creating silver- or gold-enriched areas.

The reasons why Moche metallurgists created this surface combination of metals with a fascinating, typical gold–silver duality could be religious, symbolic, spiritual or otherwise motivate.^11,15–17,25^ Some scholars hypothesize that gold could be related to the sun or masculinity and silver to the moon or femininity; therefore, the coupling between them could express symbolism related to duality.^11,15–17,25^ It is important to note that the creation of these symbolic masterpieces was made possible by sophisticated metalworking techniques and the ability to manipulate the surface chemical composition of ternary alloys allowing for significant savings of precious metal.

The distinct transition between the silver and gold areas in the *narigueras* examined (Figure 3, Figure 4) reveals no discernible morphological difference or evidence of joining two metal sheets. This finding further supports the hypothesis that repeated hammering, annealing, and pickling of a single Ag-based alloy sheet resulted in successful surface manipulation. The formation of these layers was likely accomplished by deliberately immersing the shaped *nariguera* in etching solutions or by applying poultices, thereby achieving targeted surface depletion. This method specifically involved removing copper or both copper and silver from the wetted surface (encompassing both the front and back sides). The result was an outermost silver- or gold-enriched layer that offered a captivating dual aesthetic. In addition, it is plausible that the gold or silver areas were obtained by applying different tailored pickling procedures and selecting different suitable etching materials.

BSE-FE-SEM and OM images shown in Figure 4 and Figure 7a also show minor parallel cracks within the bulk alloy, respectively. These fractures may have been formed due to impurity segregation phenomena and heat treatments.^24,25,50–52^ To prevent compromising the *nariguera*’s integrity, it is imperative to avoid mechanical stresses. Furthermore, to prevent detachment and loss of the thin gold and silver film, care must be taken during cleaning and removal of surface alteration products.

Optical microscopy investigation yielded further noteworthy information regarding the identification of copper corrosion products. The dark-field image (Figure 6b and Figure 6c) shows an external, Ag-enriched layer approximately 3 μm thick (front) and a thin layer of reddish cuprite (Cu_2_O) beneath the Cu-depleted region. A surface enrichment of silver with a granular structure was also observed on the *nariguera*’s back.

To obtain additional microchemical and morphological insights into the surface manipulation processes, we also examined the *nariguera*’s unburnished silver reverse, also considering its observed granular-like morphology. The results of the BSE-FE-SEM and EDS analyses are reported in Figure 8b–f. These data, to be compared with the findings from the front side shown in Figure 5, reveal metallic silver curls formed by aggregates of filiform silver fibers with widths ranging from less than 1 to approximately 12 μm. The Ag fiber size is generally nonuniform along their length, largest at the base and smallest at the end (Figure 8b–f). These silver curls could be related to the surface etching process used to remove copper from the ternary alloy surface, as described above. This process induces surface diffusion of silver atoms along the step edges and their continuous accumulation and rearrangement through self-organization, potentially leading to the growth of a silver epitaxial structure.^49,50,53^ The presence of filiform silver on the *nariguera*’s back reveals that this surface was not mechanically burnished, unlike the anterior side, where the silver enrichments were flattened, likely fragmented and gently compressed with appropriate metal, bone, or stone tools, producing a smooth, shiny, and aesthetically pleasing surface comparable to solid silver.^3,4,25,26^

**Figure 8:**
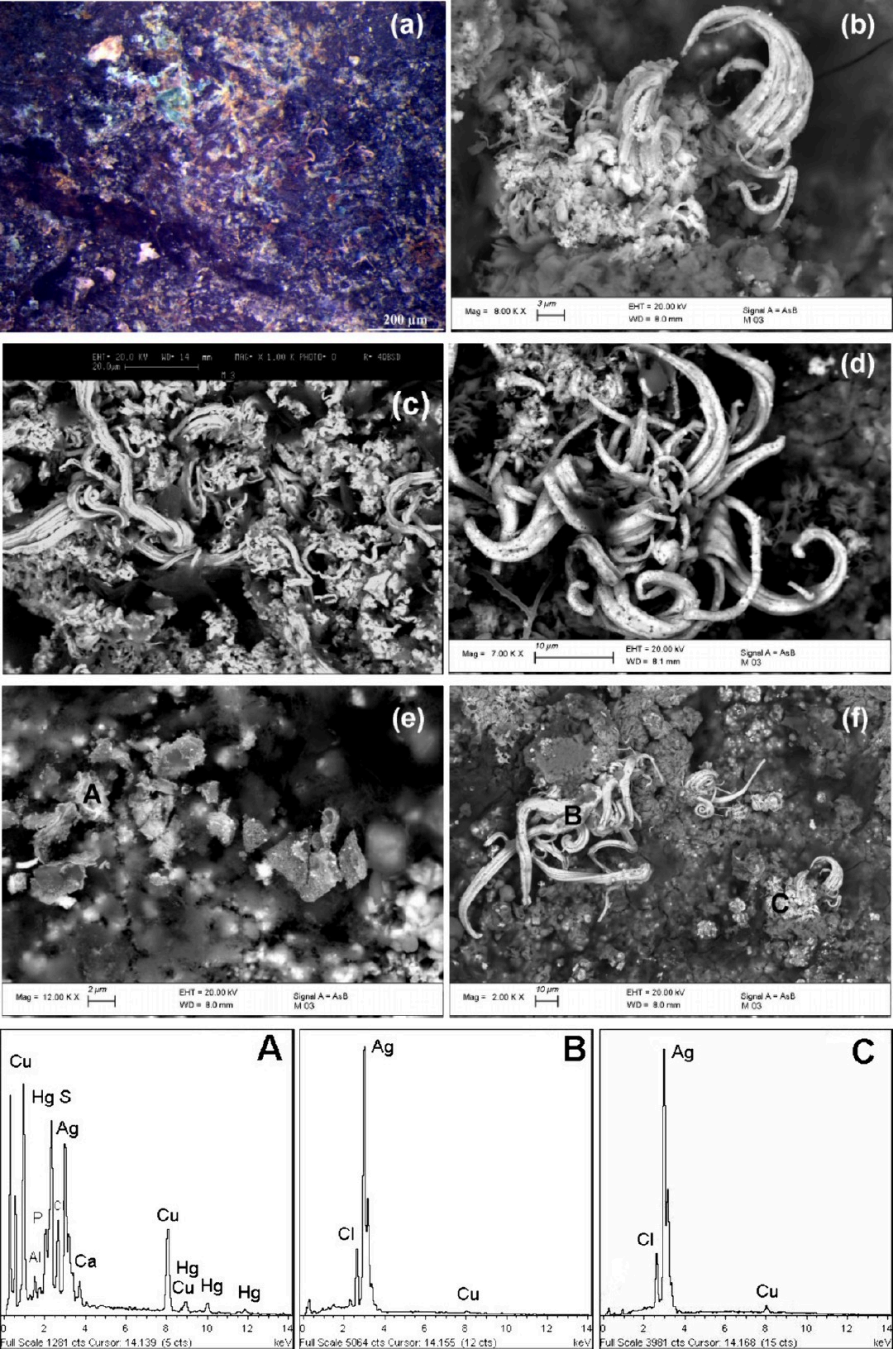
OM image (a) and BSE-FE-SEM micrographs (b)–(f) showing the morphology of the back side. The local chemical composition is revealed by EDS spectra A–C. The comparison with the images shown in Figure 5 evidences the surface micromorphological differences between front and back side.

The chemical composition of the silver wires is shown by the EDS spectra A–C reported in Figure 8. Semiquantitative analysis, with an approximate detection limit of about 0.1 wt %, indicates that the fibers’ silver content is over 95 wt %. The EDS analysis has also revealed a small amount of chlorine, probably related to the formation of a small amount of chlorargyrite (AgCl) due to the surrounding Cl^–^ containing environment or residues of pickling compounds.^2,4,25^

The fibrous silver formed on the *nariguera*’s surface exhibits a morphology strikingly similar to the architecture observed in natural metallic silver curls.^54–56^ These formations are distinguished by a polycrystalline, face-centered-cubic microstructure frequently associated with twinning. The native silver wires have an anisotropic shape and appear to be formed by the elongation and stacking of planar crystals. Anderson and colleagues observed that the morphology of natural silver wires is consistent with rapid basal addition of Ag atoms confined with a lateral growth footprint, supporting the role of solid-state ion conduction (SSIC) in forming natural and synthetic silver wires.^55–57^

Finally, as previously mentioned, BSE-FE-SEM and EDS analysis (Figure 8e, EDS spectrum A) revealed minute micrometric granules containing mercury, invariably associated with sulfur. The presence of this mineral species is linked to the use of red cinnabar (HgS) in powder form to sprinkle on the deceased or to decorate accompanying grave goods.^7,8,20,43^ This practice has been thoroughly documented in the Lady of Cao’s tomb and EDS analysis of certain objects, including a gold bead (Figure S1). This assertion is further supported by a cinnabar layer observed on other artifacts unearthed in the Lords of Sipan tombs, including a gilded semicircular diadem found in the Tomb 14 (Figure S2). The presence of mercury could be misleading, as it might be assumed it was used to join Ag and Au plates or to gild the examined *nariguera*.^27–29,58–61^ However, scholars have reported that the use of amalgam for gilding or soldering in preconquest Peru has never been substantiated.^13,14,17–22,24,25^

## Concluding Remarks and Future Perspective

This study aims to examine in detail the surface and bulk structure of captivating pre-Columbian metal artifacts from the Lady of Cao’s tomb (midfifth century AD). We particularly emphasize the remarkable ceremonial pieces crafted for an elite, signifying power and status. A golden *atlatl* and the nose jewelry *nariguera* with its fascinating bimetallic gold and silver appearance, serves as a prime example.

Our approach, based on an integrated group of analytical techniques, revealed the use of surface manipulation to create artifacts coated with adherent, micrometric films of silver and gold, sometimes even on the same object.

These findings lay a foundation for understanding aspects of empirical, yet sophisticated, pre-Columbian metallurgy in South America. They also demonstrate the extraordinary skill and virtuosity of Moche artisans in using subtractive methods to create large, uniform gold or silver surfaces on Cu- and Ag-based alloys objects.

The fabrication of these surfaces depended on the availability of specific chemical compounds and metals coupled with the dexterity of the Moche metalworkers. This enabled the production of specific ternary alloys and the selection of appropriate materials for surface manipulation.

Such empirical skills allowed for the tailored chemical modification of the surface, involving the selective removal of copper or copper and silver from the outermost layers. This process left an *in situ* silver or gold enrichment with a distinctive appearance, likely imbued with symbolic or shamanic values.^25^ It is noteworthy that this unique ability to manipulate matter at the micro- and nanoscale, combined with the goldsmiths’ artistic creativity, enabled the creation of fascinating artworks.

Moreover, the results of this study could inspire materials scientists to engineer innovative materials, potentially leading to the development of a broad array of novel surface micro- and nanostructures with distinct characteristics. These include the favorable mechanical properties of the Ag–Cu substrate and the enhanced thermal and electrical conductivity of the surface.^62,63^

From a conservation perspective, the findings help us to safely clean and treat the surfaces using nontoxic, long-lasting materials, possibly based on emerging nanotechnologies. We also recommend that artifacts be maintained in a humidity- and sulfide-free atmosphere and that dangerous mechanical stresses be avoided.^33–35,50–52^

## Supplementary Material



## Notes

Section 1, BSE-FE-SEM, BSE-SEM and OM images, EDS spectra and XRD pattern for Moche artifacts decorated with cinnabar (HgS); Section 2, methods and experimental details (PDF)
