# Supplementary material for: Surface Manipulation in Cu- and Ag-Based Pre-Columbian Artifacts
Source: Acc Chem Res. 2025 Sep 16;58(19):2997–3009. doi: 10.1021/acs.accounts.5c00415 (PMC12509263; doi:10.1021/acs.accounts.5c00415)
Supplement: Supplementary file 1 [file ar5c00415_si_001.pdf]

Supporting information for

Surface manipulation in Cu- and Ag-based pre-Columbian artefacts

Gabriel Maria Ingo*, Cristina Riccucci, Francesca Boccaccini, Marianna Pascucci, Elena Messina, and Gabriella Di Carlo

Istituto per lo studio dei materiali nanostrutturati, Consiglio Nazionale delle Ricerche, Strada Provinciale 35d, n. 9, 00010, Montelibretti, Rome, Italy

* Corresponding author: gabrielmaria.ingo@cnr.it (G.M.I.)

- **Section 1: BSE-FE-SEM, BSE-SEM and OM images, EDS spectra and XRD results for Moche artifacts decorated with cinnabar (HgS).**
- **Section 2: Methods and experimental details**

<u>Section 1</u>

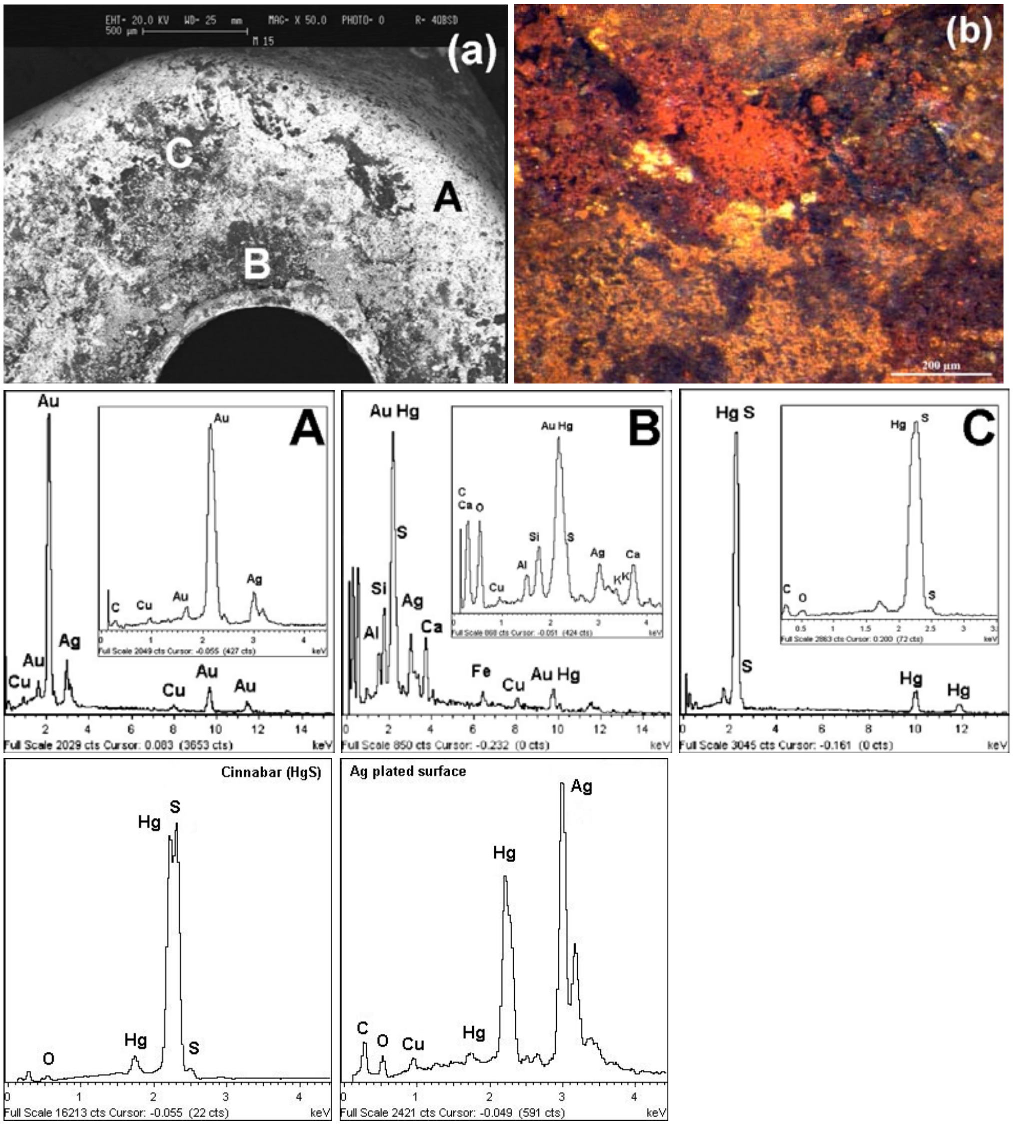

Figure S1 BSE-SEM micrograph (a) and OM image (b) of a gold bead found in the tomb of the Lady of Cao. The presence of red cinnabar (HgS) is revealed by the EDS spectra B and C where the enlargement of the energy range from 0 to about 4 keV is reported to better reveal the spectral features of the Hg, Au and S signals. For comparison, the EDS spectra of pure cinnabar and an archaeological silver-plated object coated with Ag-Hg amalgam are also shown in the third row.

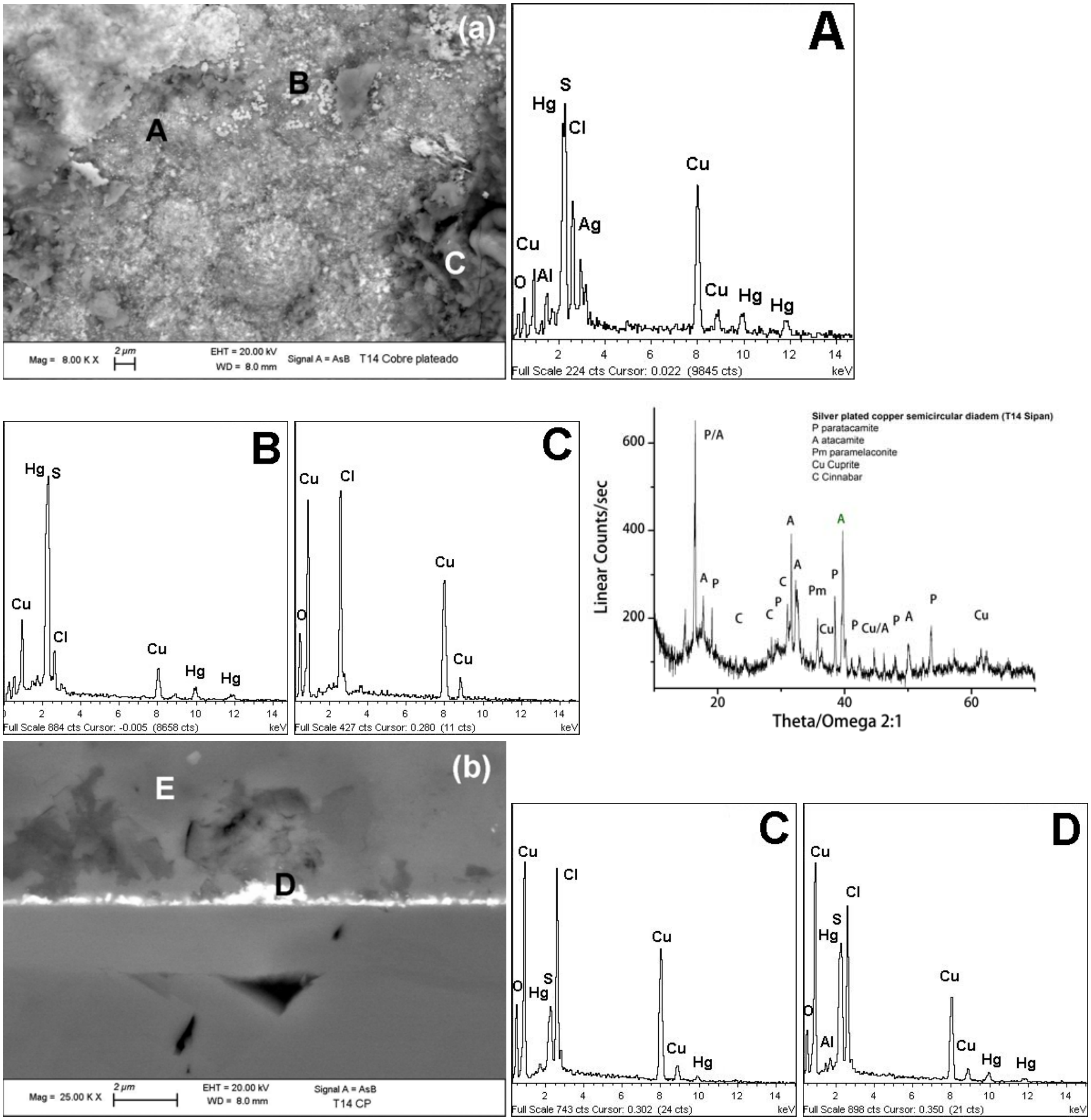

Figure S2 BSE-FE-SEM image (a), EDS spectra, and XRD pattern of the surface of a silver-plated semicircular copper diadem, almost completely mineralized, found in the Moche tomb 14 of the Lords of Sipán, Peru (AD 250). EDS spectra A and B indicate that the object was coated with cinnabar (HgS), a finding confirmed by the XRD pattern. The use of HgS is further supported by the BSE-FE-SEM image (b) and EDS spectra C and D of cross-sectioned *patina* naturally grown on the artifact, which has retained a thin residual film of the HgS layer appearing white in the BSE-FE-SEM image (b).

Section 2

Archaeological objects

The artifacts and small fragments were selected by the archaeologists of the Conservation Laboratory of the Lady of Cao Museum with the aim of increasing the information on the chemical composition and structure of the artifacts unearthed in the tomb of the Lady of Cao. The selected objects were preliminarily studied *in situ* by

optical microscopy (OM) and then in the laboratories by means of FE-SEM-EDS, XRD, XPS and OM techniques. The samples investigated were fragments of a *nariguera*, an *atlatl* , other gilded plates and some small objects as a gold bead. Other samples examined were small fragments accidentally detached from the objects and small amounts of external corrosion products removed from the *patina* of the objects. The artifacts or the fragments were first examined in the state in which they were received, and then some representative samples were carefully sectioned in order to study in details the nature and thickness of the precious metal thin layers, as well as the alloy composition and bulk structure The preparation of the sectioned samples began with the embedding of the fragments in a two-component resin, characterized by a curing time of 24 hours, and continued with a careful sectioning carried out with a diamond saw in order to preserve the micro-structural features and, in particular, of the outermost region. The final treatment was carried out using silicon carbide paper up to 1200 grit with a final polishing using various diamond pastes up to 0.1 μm and using ethanol instead of water to prevent the solubilization of the corrosion compounds. The samples to be observed by high spatial resolution field emission scanning electron microscopy (FE-SEM) and scanning electron microscopy (SEM) and analyzed by energy dispersive spectroscopy (EDS), were coated with carbon using a carbon sputter coater, a Bal-Tech SCD 500 (working pressure $5x10^{-3}$ mbar) to obtain a conductive carbon thin film. Finally, a suitable carbon tape was to hold the samples, also to avoid charging effects induced on the sample by the electron beam.

Optical and scanning electron microscopy-energy dispersive spectroscopy analyses

The archaeological fragments as received state and the sectioned samples were examined using various optical microscopes (Leica MZFL III stereomicroscope, Leica Application Suite multifocus stereomicroscope and Leica MEF IV metallographic microscope) to study morphological and metallurgical features. In addition, at the Museum of the Lady of Cao (Complejo Arqueológico El Brujo, Peru), a Zeiss optical microscope equipped with a digital camera was used to examine archaeological artifacts *in situ*.

The fragments of the artifacts and the cross-sectioned samples were investigated by using a high spatial resolution field emission scanning electron microscopy (FE-SEM) LEO Gemini 1530 (Zeiss, Germany) and a scanning electron microscopy (SEM) Stereoscan 360 with a thermoionic electron gun fitted with a LaB_6 filament cathode (Cambridge, UK), equipped with an EDS INCA 450 and an EDS INCA 250 systems (Oxford Instruments Analytical, UK), respectively. The microscopes were equipped with secondary electron (SE) and four-sectors back-scattered electron (BSE) detectors. The SE system captures secondary electrons to primarily reveal surface topography, while the BSE system collects back-scattered electrons offering contrast based on atomic number that reveals compositional variations within the sample. The energy was set at an accelerating voltage of 20 kV for the SEM investigation, while the FE-SEM characterization was performed by varying the voltage from 3 kV to 20 kV.

X-ray diffraction (XRD) investigation

The XRD characterization was carried out by investigating the artifact surfaces and the powdered materials taken from the objects by using a Siemens 5000 X-ray powder diffractometer with Ni-filtered Cu K_α radiation (λ = 0.154056 nm). The experimental parameters were: angular values from 10° to 90° in additive mode, step size 0.05° and sampling time of 20 s. The XRD results were studied by using specific databases and literature information.

X-ray photoelectron spectroscopy (XPS)

XPS measurements were carried out using an ESCALAB Mk II spectrometer equipped with a hemispherical electron energy analyzer and a double anode X-ray source (unmonochromatic Al Kα and Mg Kα radiation, 1486.6 eV and 1253,6 eV, respectively). The accuracy of the binding energy (BE) was measured to be ± 0.1 eV. The BE values of the Sn $3d_{5/2}$ peak from cassiterite (SnO_2), the Zr $3d_{5/2}$ peak from zirconia (ZrO_2) and of the Au $4f_{7/2}$ signal from Ar^+-cleaned Au 99.99% were 487.0 eV, 182.4 eV and 84.0 eV, respectively.[40,64] In order to avoid, or at least reduce, any potential sample damage caused by X-ray irradiation, short acquisition times were adopted and a copper sample holder cooled by liquid N_2[41] was used. Further experimental details are reported elsewhere.[1,25-26]

REFERENCES

(64) Ingo, G.M. & Padeletti, G. Segregation aspects at the fracture surfaces of 8 wt% yttria-zirconia thermal barrier coatings. *Surf. Interface Analysis* **21**, 450-454 (1994).
